# Standardised data collection in prehospital critical care: a comparison of medical problem categories and discharge diagnoses

**DOI:** 10.1186/s13049-022-01013-5

**Published:** 2022-04-12

**Authors:** Miretta Tommila, Jukka Pappinen, Lasse Raatiniemi, Anssi Saviluoto, Tuukka Toivonen, Johannes Björkman, Jouni Nurmi

**Affiliations:** 1 Department of Perioperative Services, Intensive Care Medicine and Pain Management, Turku University Hospital and University of Turku, Turku, Finland; 2 FinnHEMS Ltd, HEMS Operations, Vantaa, Finland; 3 Centre for Prehospital Emergency Care, Oulu, Finland; 4 Research Group of Surgery, Anaesthesiology and Intensive Care, Division of Anesthesiology Oulu University Hospital, Medical Research Centre, University of Oulu, Oulu, Finland; 5 University of Eastern Finland, Kuopio, Finland; 6 Emergency Medicine and Services, Helsinki University Hospital and University of Helsinki, FinnHEMS 10, Vesikuja 9, 01530 Vantaa, Finland

**Keywords:** Air ambulances, Emergency medical services, Critical care, Medical problem reporting, Prehospital, Documenting

## Abstract

**Background:**

Prehospital medical problem reporting is essential in the management of helicopter emergency medical services (HEMS) operations. The consensus-based template for reporting and documenting in physician-staffed prehospital services exists and the classification of medical problems presented in the template is widely used in research and quality improvement. However, validation of the reported prehospital medical problem is lacking. This study aimed to describe the in-hospital diagnoses, patient characteristics and medical interventions in different categories of medical problems.

**Methods:**

This retrospective, observational registry study examined the 10 most common in-hospital International Statistical Classification of Disease (ICD-10) diagnoseswithin different prehospital medical problem categories, defined by the HEMS physician/paramedic immediately after the mission was completed. Data were gathered from a national HEMS quality registry and a national hospital discharge registry. Patient characteristics and medical interventions related to different medical problem categories are also described.

**Results:**

A total of 33,844 patients were included in the analyses. All the medical problem categories included a broad spectrum of ICD-10 diagnoses (the number of diagnosis classes per medical problem category ranged from 73 to 403). The most frequent diagnoses were mainly consistent with the reported medical problems. Overlapping of ICD-10 diagnoses was mostly seen in two medical problem categories: stroke and acute neurology excluding stroke. Additionally, typical patient characteristics and disturbances in vital signs were related to adequate medical problem categories.

**Conclusions:**

Medical problems reported by HEMS personnel have adequate correspondence to hospital discharge diagnoses. However, the classification of cerebrovascular accidents remains challenging.

## Introduction

Documenting and reporting are important parts of prehospital care. Conscientious documenting is a legal necessity as a part of a patient’s treatment, but it is also an important way to acquire data for clinical quality improvement and benchmarking and for research purposes. Certainly, medical problem categorisation is a prerequisite in the management of helicopter emergency medical services (HEMS) operations. Designed to standardise prehospital documenting and reporting policies, a consensus-based template for physician-staffed prehospital services was first published in 2011 [1]. The feasibility of this template has been demonstrated, and it has been recently updated [2, 3]

Despite the existing international template, there are still variations in the ways patients’ medical problems are reported in different HEMS systems. In Nordic HEMS, the reporting rests on the principles of the international template, although each system has its own modifications [4–6]. In comparison to other European services, there is even more divergence in reporting of medical problems [7, 8]. The categories in the consensus template are widely used in research reports, for example in studies regarding airway management [9] and physiological disturbances [10] in prehospital critical care. However, the differences in reporting make it challenging to compare these services. Moreover, the lack of data on exact medical conditions in each category makes it challenging to generalise the findings.

Currently, validation of the reported prehospital medical problem is lacking.The parity between medical problem categorisation and in-hospital diagnoses is unknown.. Moreover, it is not known if the categorisations made by providers in the field are accurate. In the current study, we aimed to describe the in-hospital diagnosis classes and patient characteristics of the patients classified by HEMS personnel into different medical problem categories, according to the consensus template of reporting physician-provided prehospital care [1]. Furthermore, we introduce patient characteristics, medical interventions and 30-day mortality associated with each medical problem category in the Finnish HEMS.

## Methods

### Study design

We performed a retrospective, observational registry study describing the 10 most common International Statistical Classification of Diseases (ICD-10) diagnoses within medical problem categories as defined in the consensus-based template for physician-staffed prehospital services [1]. Additionally, we examined typical patient characteristics and medical interventions related to different medical problem categories. Ethical permission was granted by the Ethical Board of the University of Helsinki (HUS/3115/2019 §194). Study permission was granted by all the participating hospital districts. The reporting of the study adheres to Strengthening the Reporting of Observational studies in Epidemiology (STROBE) guidelines [11].

### Setting

The Finnish HEMS system consists of five physician-staffed units and one paramedic-staffed unit. The paramedic-staffed unit serves the vast and sparsely populated Lapland. The units are primarily dispatched to emergencies by the Emergency Response Centres, with slight regional differences in the dispatch criteria. The dispatch criteria, mission profile and the national HEMS have recently been described elsewhere [6].

### Participants

All patients encountered by HEMS units between 1 January 2012 and 31 December 2018 were included in the study. The timeframe depicts the foundation of the database and the latest practical point. Due to the study’s retrospective design, no power calculation was performed and all available data were included.

### Data sources

#### FinnHEMS database (FHDB)

The FHDB is a national HEMS quality registry containing details on all HEMS missions in Finland. After each mission, the HEMS crew enters the mission details into the database. There have been no changes related to reporting of medical problem categorisation or other investigated parameters during the study period. FHDB and data collection have recently been described in detail in previous papers [6, 12].

#### The national hospital discharge register (HILMO)

The HILMO database is maintained by the Finnish Institute for Health and Welfare. All organisations providing inpatient care are required by law to enter data on all hospital admissions and discharge. Upon discharge, up to five main diagnoses—following the ICD-10 nomenclature—are then entered into the HILMO register. The entered data also include additional information, such as provided treatment (Nordic Classification of Surgical Procedures [NCSP]) and length-of-stay. The completeness and accuracy of the HILMO register have been evaluated to be appropriate, and the validity of the register has been stated to be suitable for health services research [13]. For our study, all the main diagnoses from consecutive hospital admissions between 1 January 2012 and 31 December 2018 were acquired from the HILMO database.

#### Finnish digital and population data service agency

Information on living status or date of death was acquired for all the encountered patients from the Finnish Digital and Population Data Service Agency until the end of October 2019.

### Variables

In the FHDB, each patient is assigned one of 10 possible mission medical problems, according to the HEMS provider’s assessment on the true medical condition after the mission is completed. The medical problem categories, as defined in the consensus-based template for physician-staffed prehospital services, are: cardiac arrest, trauma, breathing difficulties, chest pain, stroke, acute neurology excluding stroke, psychiatry including intoxication, obstetrics and childbirth, infection, and other [1].

Outcome was defined as the main diagnosis classes at hospital discharge, extracted from the HILMO register. Since the discharge diagnoses might not be arranged in order of importance, all the main diagnoses were included. The diagnoses are reported as the ICD-10 main categories, i.e. without numbers after the decimal separator (for example, I21 corresponding to acute myocardial infarction, including subheadings, e.g. I21.1 for acute transmural myocardial infarction of anterior wall).

In addition to the hospital discharge diagnoses, we gathered typical patient characteristics categorised by each medical problem as follows: sex, age, first vital parameter values recorded by HEMS personnel (oxygen saturation, heart rate, systolic blood pressure, and Glasgow Coma Score [GCS]). We calculated the proportion of hypoxic and hypotensive patients per every medical problem category. The definition of hypoxia was an oxygen saturation below 90%; hypotension was defined as systolic blood pressure under 90 mmHg [14]. We also determined the proportion of patients treated with advanced airway management techniques used or with vasoactive medication given within each category. Finally, we assessed 30-day mortality in different medical problem categories.

### Statistical methods

Quantitative variables are presented as means with standard deviation (± SD) or median with the interquartile range (25th–75th percentile), depending on the distribution. Categorial variables are presented as n (%). Missing data were not included in the analysis; however, the percentages of missing data are reported as n (%). We used IBM SPSS Statistics 25 (IBM Corporation, Armonk, NY, USA) for all statistical analyses.

## Results

**Fig. 1:**
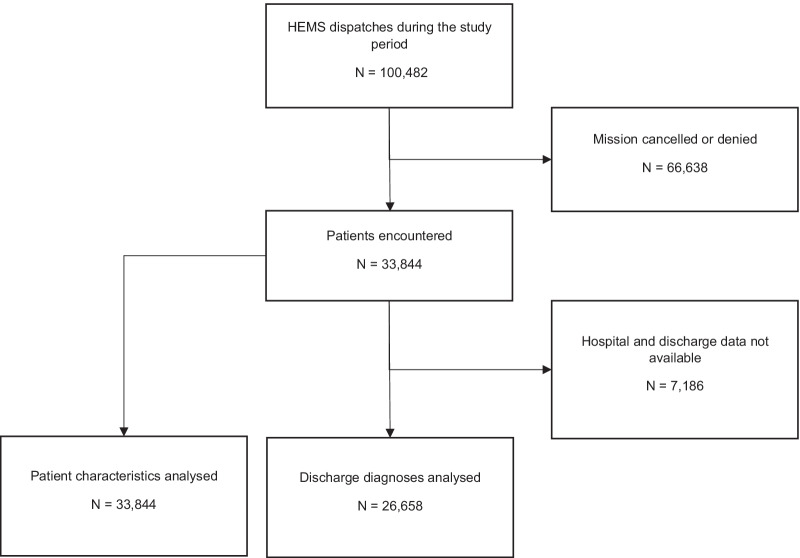
Patient selection flow chart

A total of 33,844 patients were recorded in the FHDB as encountered by HEMS personnel within the study timeframe. All these patients were included in the study (Fig. 1). Due to the possibility of several ICD-10 diagnoses for each individual patient, the total number of ICD-10 diagnoses was 36,483. The number of diagnoses under each medical problem category ranged from 73 to 403, and the proportion of the diagnoses not covered by the 10 most frequent diagnoses varied 16–67% between the categories.

The 10 most frequent diagnoses under each specific medical problem category are described in Table 1. There was a vast heterogeneity of ICD-10 diagnoses in every medical problem category, but the diagnoses were mainly consistent with the reported medical problem. The greatest variation was noticed under the medical problem category of stroke, in which that category was mixed with the acute neurology excluding stroke category. Among the more typical diagnoses in the medical problem category of cardiac arrest, non-traumatic subarachnoid haemorrhage was ranked fifth. Furthermore, intracranial injury represented 25% of the ICD-10 diagnoses categorised under trauma.

**Table 1:** Ten most frequent ICD-10 top level diagnoses categorized by medical problem. Presented as patient category (number of separate top-level diagnoses) or *n* (%)

Diagnoses	N	%	Diagnoses	N	%
Cardiac arrest (238 diagnosis blocks in total)			Trauma (368)		
Cardiac arrest	1904	44.1	Intracranial injury	2455	24.6
Myocardial infarction	583	13.5	Fracture of rib(s), sternum and thoracic spine	655	6.6
Ill-defined and unknown cause of mortality	324	7.5	Fracture of skull and facial bones	480	4.8
Other cardiac arrhythmias	172	4.0	Fracture of lumbar spine and pelvis	461	4.6
Nontraumatic subarachnoid hemorrhage	98	2.3	Injury of other and unspecified intrathoracic organs	380	3.8
Chronic ischemic heart disease	95	2.2	Open wound of head	375	3.8
Other disorders of brain	53	1.2	Fracture of lower leg, including ankle	284	2.8
Pulmonary embolism	46	1.1	Fracture of femur	270	2.7
Heart failure	39	0.9	Fracture of neck	247	2.5
Pneumonia, unspecified organism	37	0.9	Injury of intra-abdominal organs	224	2.2
Other diagnoses	963	22.3	Other diagnoses	4144	41.5
Breathing difficulties (273)			Chest pain (123)		
Heart failure	403	14.5	Myocardial infarction	749	42.2
Pneumonia, organism unspecified	347	12.5	Pain in throat and chest	243	13.7
Other chronic obstructive pulmonary disease	212	7.6	Angina pectoris	173	9.7
Abnormalities of breathing	156	5.6	Chronic ischemic heart disease	93	5.2
Acute myocardial infarction	133	4.8	Atrial fibrillation and flutter	75	4.2
Other respiratory disorders	92	3.3	Paroxysmal tachycardia	46	2.6
Bacterial pneumonia, not elsewhere classified	75	2.7	Heart failure	40	2.3
Adverse effects, not elsewhere classified	65	2.3	Syncope and collapse	23	1.3
Asthma	56	2.0	Dissection of aorta	20	1.1
Foreign body in respiratory tract	54	1.9	Abdominal and pelvic pain	18	1.0
Other diagnoses	1188	42.7	Other diagnoses	295	16.6
Stroke (191)			Other neurological (332)		
Cerebral infarction	724	22.0	Convulsions, not elsewhere classified	994	17.9
Nontraumatic intracerebral hemorrhage	538	16.3	Epilepsy and recurrent seizures	994	17.9
Nontraumatic subarachnoid hemorrhage	492	14.9	Status epilepticus	363	6.5
Epilepsy and recurrent seizures	153	4.6	Nontraumatic intracerebral hemorrhage	289	5.2
Intracranial injury	143	4.3	Mental and behavioural disorders due to use of alcohol	245	4.4
Convulsions, not elsewhere classified	142	4.3	Personality disorder	237	4.3
Transient cerebral ischemic attacks and related syndromes	109	3.3	Syncope and collapse	210	3.8
Somnolence, stupor and coma	77	2.3	Cerebral infarction	204	3.7
Syncope and collapse	71	2.2	Somnolence, stupor and coma	181	3.3
Status epilepticus	53	1.6	Intracranial injury	175	3.2
Other diagnoses	796	31.8	Other diagnoses	1663	29.9
Psychiatric including intoxication (220)			Gynecologic or obstetric (73)		
Poisoning or adverse effect of medicament	1348	37.8	Full-term uncomplicated delivery	368	47.4
Mental and behav. disorders due to alcohol	556	15.6	Maternal postpartum care and examination	46	5.9
Depression	137	3.8	Supervision of normal pregnancy	45	5.8
Poisoning due to narcotics or hallucinogens	122	3.4	False labor	43	5.5
Toxic effect of alcohol	99	2.8	Single delivery by caesarean section	34	4.4
Somnolence, stupor and coma	81	2.3	Abdominal and pelvic pain	22	2.8
Recurrent major depression	53	1.5	Obstetrical hemorrhage	17	2.2
Opioid related disorders	49	1.4	Single delivery by forceps and vacuum extractor	14	1.8
Other psychoactive substance related disorders	49	1.4	Placenta previa	14	1.8
Convulsions, not elsewhere classified	46	1.3	Premature rupture of membranes	13	1.7
Other diagnoses	1030	28.9	Other diagnoses	160	20.6
Infection (146)			Oher (403)		
Other sepsis	84	14.6	Syncope and collapse	205	5.3
Pneumonia, unspecified organism	71	12.3	Paroxysmal tachycardia	162	4.2
Convulsions, not elsewhere classified	50	8.7	Dissection of aorta	131	3.4
Bacterial infection of unspecified site	35	6.1	Hypothermia	127	3.3
Bacterial pneumonia, not elsewhere classified	15	2.6	Other diseases of digestive system	124	3.2
Infectious gastroenteritis and colitis, unspecified	14	2.4	Poisoning or adverse effect of medicament	122	3.2
Erysipelas	11	1.9	Mental and behav. disorders due to alcohol	118	3.1
Streptococcal sepsis	10	1.7	Adverse effects, not elsewhere classified	115	3.0
Acute tubulo-interstitial nephritis	9	1.6	Atrial fibrillation and flutter	92	2.4
Abdominal and pelvic pain	9	1.6	Abdominal and pelvic pain	90	2.3
Other diagnoses	268	46.5	Other diagnoses	2577	66.7

The characteristics of the patients in different categories are presented in Table 2. The patients in the trauma, psychiatric including intoxication, and obstetrics and childbirth categories were, on average, younger than the patients in the other medical problem categories. The lowest values of GCS were found in the cardiac arrest, stroke, acute neurology excluding stroke and psychiatric including intoxication categories.

**Table 2:** Patient characteristics categorized by medical problem. Presented as N, proportion (%) or median (25th–75th percentile)

Medical problem	N	Sex; male (%)	Age (years)		Oxygen saturation (%)		Heart rate (1/min)		Systolic blood pressure (mmHg)		GCS	
Cardiac arrest	6900	71.8	68	58–78	*		*		*		3	3–3
Trauma	8897	71.8	40	23–59	98	95–99	90	80–105	130	115–150	15	12–15
Breathing difficulties	2077	53.9	69	52–80	90	80–96	108	90–126	140	118–169	15	13–15
Chest pain	1380	68.0	68	60–78	97	95–98	80	65–100	140	112–162	15	15–15
Stroke	2295	50.5	72	61–80	97	94–98	83	70–100	157	133–185	8	4–13
Acute neurology (excluding stroke)	4366	57.3	58	32–72	97	94–98	98	80–119	134	113–158	9	5–14
Psychiatric (including intoxication)	3318	55.1	37	25–50	97	94–99	90	75–105	120	105–137	8	3–13
Gynecologic or obstetric	689	1.9	30	26–35	98	97–99	90	80–100	125	115–139	15	15–15
Infection	422	56.6	60	19–75	95	90–98	110	90–130	110	90–130	14	9–15
Other	3500	63.0	62	42–76	97	94–99	90	70–112	120	96–143	15	15–15

All values are first values recorded by HEMS

GCS: Glasgow coma score

HEMS: Helicopter emergency medical services

^*^Initial values are not reported as marked proportion of the patients are encountered with cardiac arrest and unmeasurable vital signs

The prevalence of hypoxia and hypotension as well as interventions performed in different patient categories are presented in Table 3. Hypoxia was most common in the breathing difficulties and infection categories. Hypotension was most frequently noticed in the infection category and the other diagnoses category.

**Table 3:** Performed medical interventions, 30-day mortality and proportions of patients suffering from hypoxia or hypotension when encountered by HEMS. Reported as percentage (%)

Medical problem	Hypoxic (%)	Advanced airway (%)	Hypotensive (%)	Vasoactive medication (%)	30-day mortality (%)
Cardiac arrest	*	49	*	59	79
Trauma	6	17	6	11	11
Breathing difficulties	47	11	7	23	18
Chest pain	8	3	12	34	8
Stroke	9	38	3	24	38
Acute neurology (excluding stroke)	11	25	6	14	13
Psychiatric (including intoxication)	11	25	11	12	2
Gynecologic or obstetric	0.8	1	3	0.4	0.2
Infection	20	13	24	26	18
Other	9	11	19	18	14

^*^Initial values cannot be measured

Vasoactive medication was most often used for the patients in the cardiac arrest, breathing difficulties, chest pain, stroke, and infection categories. Advanced airway was most common in the categories which also had the lowest GCS values. Significant variation in 30-day mortality was observed between the groups.

Patients’ medical problem and whether vasoactive medication was given were available for all patients, whereas sex and age was missing for 383 (1.1%) and 28 (0.01%) patients respectively. Out of the vital signs at the time of encounter systolic blood pressure could not be measured for 885 (2.6%) and was missing or not measured for 8492 (25%) patients. Heart rate could not be measured for 537 (1.6%) patients and was missing for 7711 (23%) patients, oxygen saturation could not be measure for 5184 (15%) and was missing for 4236 (13%) GCS was missing for 2076 (6.1%) patients. Whether an advanced airway was used was missing for 160 (0.5%), 30-day mortality was unavailable for 2079 (6.1%) and no discharge diagnoses were available for 7630 (23%) patients.

## Discussion

The main finding of this study is that the medical problems reported by HEMS personnel were comparable to the hospital discharge diagnoses, excluding the medical problem reported as stroke. Furthermore, we observed noticeable heterogeneity and a broad spectrum of the diagnoses in the patients treated by HEMS personnel.

To the best of our knowledge, this is the first study that has investigated the hospital diagnoses of the patients treated by HEMS personnel and classified according to the consensus-based reporting template. The strength of the study is its use of large, nationwide registries as a data source. A significant number of HEMS missions was included in the analysis, and the overall mission reporting quality has been reported to be at an advisable level [6, 12]. The data collection in the HEMS database is mainly based on the international guidelines [1], although with slight modifications [6].

However, our study has several limitations. As a registry study, the major limitation is its retrospective nature. A comparison of the databases is somewhat flawed: data entered into HILMO have shown excellent validity for single disease groups but lacking in, for example, polytrauma patients [15]. Moreover, according to our hypothesis, the discharge diagnosis is assumed to be correct; however, that is not always the case and not all patients receive a definitive diagnosis. Furthermore, the accuracy of the prehospital classification was not evaluated in an objective and systematic way as there is no golden standard for prehospital category for each discharge diagnosis. Thus, the conclusion of the study is subjective and needs to be interpreted according to the results obtained. The inexact nature of medical science and inter-operator differences in clinical judgement must be considered and accepted when interpreting the results of medical studies.

In our study, many of the patients diagnosed with stroke had a discharge diagnosis more suitable for the medical problem of acute neurology excluding stroke. In fact, the definition of a stroke also broadly includes intracerebral haemorrhage and subarachnoid haemorrhage in addition to an ischemic cerebral event [16]. Our finding reflects the difficulty of diagnosing different intracranial disorders and neurological emergencies solely based on clinical symptoms [17, 18]. Moreover, patients presenting classical stroke symptoms without gross impairment of consciousness are mainly treated by paramedics without HEMS assistance [19]. Hence, the stroke situations that have demanded HEMS involvement have probably been more complex.

The medical problem reported as trauma revealed that the proportion of traumatic brain injuries (TBI) is remarkably high: approximately one in four trauma patients treated by HEMS personnel suffered from a TBI. The high proportion can be explained by the fact that these patients are included in the dispatch criteria for HEMS and they have been shown to benefit from prehospital critical care [20–22].

As expected, there were no dramatic findings related to typical patient characteristics in the different medical problem categories. Sudden illness or trauma can affect people of various ages, but patients suffering trauma or psychiatric or obstetric problems seem to be younger than patients with acute cardiovascular or cerebrovascular events. Disturbances in certain vital organ functions were associated with the adequate medical problem category; for example, hypoxia was most common during respiratory distress and infections, hypotension was most common during infections and in the miscellaneous category (containing for example severe arrhythmias) and declined consciousness was common during cardiac arrest, cerebrovascular events and intoxication. Evidently, advanced airway management was most frequently associated with medical problems causing declined consciousness, but vasoactive medication was used more often not only in categories linked to hypotension, but also in patients with chest pain, stroke and breathing difficulties. However, in this study only initial values of the vital parameters are investigated, so it seems likely that patients who got vasoactive medication also presented more or less unstable hemodynamics at some stage of prehospital treatment.

The highest 30-day mortality was seen most often among cardiac arrest patients, as expected. Patients categorised under stroke had the second highest fatality. This might be explained by the fact that HEMS mainly involves in the most demanding stroke cases. Indeed, increased mortality also among patients suffering respiratory distress or infections probably reflects the severity of the typical diagnoses classified under these categories, such as serious cardiovascular events, or critical infections like sepsis or pneumonia. Furthermore, fairly notable mortality, 14%, was found in category “other”, and nearly one fifth of these patients needed also vasoactive medication. Based on these findings, this quite large group of patients seems to be severly ill, and it would be important to be able to classify these patients more thoroughly.

In addition to that, the substantial number of diagnosis groups in each patient category raises the question as to the extent to which this crude classification is appropriate in the prehospital critical care research. Adjusting outcome analyses based on this classification may not be sufficient as the groups are highly heterogenous in terms of pathophysiology. An attempt to address the effectiveness of HEMS and the prehospital critical care provided by the physicians has been made by conventional clinical studies, including cohort and case–control designs [23]. However, the intervention (prehospital critical care provided by the HEMS physician) includes many different procedures, logistic approaches, tactics, complicated clinical pathways and human factors; thus, the intervention is complex instead of simple (pharmaceutical). This complexity is increased by the significant heterogeneity of the patients being treated, as demonstrated by the current study. Thus, it is unlike to adequately summarise the evidence of the effectiveness, in general, of using conventional clinical study designs and review methods. Instead, the potential role of HEMS in treating different patient groups could be better understood using the application theories and frameworks of complex health care interventions [24].

Despite the lack of totally uniform reporting policies, our results are likely to be generalisable to other similar HEMS organisations, but for comparison to different services, for instances ambulances, more research is needed.In the future, a uniform, standardised reporting system would alleviate the comparison between different HEMS systems, both from a clinical/operational viewpoint and a scientific viewpoint.

Based on our study, it is especially important to improve the accuracy in the medical problem category of stroke. In the recently updated documenting and reporting template, this area is now divided into smaller units: stroke, acute neurology excluding stroke, reduced level of consciousness and poisoning/intoxication. This division might improve the accuracy of the classification of unconscious patients, although prehospital diagnostics discerning between stroke and other neurological emergencies is still challenging. Finally, in addition to our findings about the medical problems categories, the significant prevalence of subarachnoid haemorrhage among cardiac arrest patients must be kept in mind, and this diagnosis should be examined more closely.

## Conclusion

The medical problems categorised by HEMS personnel can be seen as valid. The greatest challenges remain in the classification of stroke and other neurological emergencies. Patients treated by HEMS personnel are characterised by significant heterogeneity and the crude classification based on the reporting template should be used cautiously in scientific research.

## Data Availability

The dataset used during the current study is available from the corresponding author upon reasonable request.

## Abbreviations

ALS: Advanced life support
FHDB: FinnHEMS database
GCS: Glasgow Coma Scale
HEMS: Helicopter emergency medical services
HILMO: National hospital discharge register
ICD-10: International statistical classification of diseases
NCSP: Nordic classification of surgical procedures
OHCA: Out-of-hospital cardiac arrest
SD: Standard deviation
STROBE: Strengthening the reporting of observational studies in epidemiology
TBI: Traumatic brain injury
